# A multifaceted implementation strategy versus passive implementation of low back pain guidelines in general practice: a cluster randomised controlled trial

**DOI:** 10.1186/s13012-016-0509-0

**Published:** 2016-10-21

**Authors:** Allan Riis, Cathrine Elgaard Jensen, Flemming Bro, Helle Terkildsen Maindal, Karin Dam Petersen, Mette Dahl Bendtsen, Martin Bach Jensen

**Affiliations:** 1 Department of Clinical Medicine, Research Unit for General Practice in Aalborg, Aalborg University, Fyrkildevej 7, 1.3, 9220 Aalborg, Denmark; 2 Danish Center for Healthcare Improvements, Aalborg University, Fibigerstræde 11, 9220 Aalborg, Denmark; 3 Research Unit for General Practice, Aarhus University, Bartholins Allé 2, 8000 Aarhus C, Denmark; 4 Department of Public Health, Aarhus University, Bartholins Allé 2, 8000 Aarhus C, Denmark; 5 Unit of Clinical Biostatistics and Bioinformatics, Aalborg University, Sdr. Skovvej 15, 9000 Aalborg, Denmark

**Keywords:** Implementation, Guidelines, Translational Medical Research, Referral and consultation, Low back pain, General practice

## Abstract

**Background:**

Guidelines are often slowly adapted into clinical practice. However, actively supporting healthcare professionals in evidence-based treatment may speed up guideline implementation. Danish low back pain (LBP) guidelines focus on primary care treatment of LBP, to reduce referrals from primary care to secondary care. The primary aim of this project was to reduce secondary care referral within 12 weeks by a multifaceted implementation strategy (MuIS).

**Methods:**

In a cluster randomised design, 189 general practices from the North Denmark Region were invited to participate. Practices were randomised (1:1) and stratified by practice size to MuIS (28 practices) or a passive implementation strategy (PaIS; 32 practices). Included were patients with LBP aged 18 to 65 years who were able to complete questionnaires, had no serious underlying pathology, and were not pregnant. We developed a MuIS including outreach visits, quality reports, and the STarT Back Tool for subgrouping patients with LBP. Both groups were offered the usual dissemination of guidelines, guideline-concordant structuring of the medical record, and a new referral opportunity for patients with psycho-social problems. In an intention-to-treat analysis, the primary and secondary outcomes pertained to the patient, and a cost-effectiveness analysis was performed from a healthcare sector perspective. Patients and the assessment of outcomes were blinded. Practices and caregivers delivering the interventions were not blinded.

**Results:**

Between January 2013 and July 2014, 60 practices were included, of which 54 practices (28 MuIS, 26 PaIS) included 1101 patients (539 MuIS, 562 PaIS). Follow-up data for the primary outcome were available on 100 % of these patients. Twenty-seven patients (5.0 %) in the MuIS group were referred to secondary care vs. 59 patients (10.5 %) in the PaIS group. The adjusted odds ratio (AOR) was 0.52 [95 % CI 0.30 to 0.90; *p* = 0.020]. The MuIS was cost-saving £−93.20 (£406.51 vs. £499.71 per patient) after 12 weeks. Conversely, the MuIS resulted in less satisfied patients after 52 weeks (AOR 0.50 [95 % CI 0.31 to 0.81; *p* = 0.004]).

**Conclusions:**

Using a MuIS changed general practice referral behaviour and was cost effective, but patients in the MuIS group were less satisfied. This study supports the application of a MuIS when implementing guidelines.

**Trial registration:**

ClinicalTrials.gov, NCT01699256

**Electronic supplementary material:**

The online version of this article (doi:10.1186/s13012-016-0509-0) contains supplementary material, which is available to authorized users.

## Background

Low back pain (LBP) is a major cause of disability and a burden for patients. For example, in the UK, LBP and neck pain are the leading causes of disability-adjusted life years [1]. The precise aetiology underlying most cases of LBP is unknown; however, biological, psychological, and social factors may all be important [2–4]. Therefore, LBP treatment may be complex, and enhancing healthcare professionals’ opportunities and competences is important to offer the best available treatment. Current evidence for LBP treatment is synthesised in guidelines to assist general practitioners (GPs) and other healthcare professionals in treating LBP and guide referrals to secondary care and supplementary treatment. However, the implementation of guidelines into practice is often slow. Therefore, knowledge regarding how to support guideline implementation is needed [5]. In 2012, a new LBP guideline was published in the North Denmark Region which focused on the roles and responsibilities of primary healthcare providers. Treatment within the first 8 weeks of the initial consultation for LBP was considered particularly important for improving primary care, thereby reducing unnecessary referrals to secondary care and consequently minimising healthcare costs [6]. Traditionally, regional guidelines have been disseminated using mostly passive implementation strategies, although passive diffusion of innovation is not generally the recommended method to change clinical behaviour [7]. Rather, supporting GPs in the uptake of LBP guidelines by multifaceted implementation strategies is more likely to generate a change in clinical behaviour [8]. In this study, we investigated whether a multifaceted implementation strategy (MuIS) was more effective in changing general practice behaviour than a passive implementation strategy (PaIS). The aim was to reduce general practices’ referrals of LBP patients to secondary healthcare within 12 weeks.

## Methods

### Study design and participants

This study was a two-armed, cluster randomised controlled trial comparing MuIS and PaIS in the North Denmark Region. The study protocol is published with open access [9]. Interventions were delivered at the practice level. We based our allocation on cluster randomisation at the practice level instead of the practitioner level because patients could be seen by different GPs and other healthcare professionals at the same general practice. General practices in the North Denmark Region were eligible for inclusion. The latest updated list of practices in the region was from October 2011. The 191 practices and 332 GPs on this list were considered eligible for inclusion. The total number of patients in the region was 579,829 with an average of 3035 listed patients per practice [10]. Excluded were practices participating in pre-testing the intervention and practices without an electronic data capture module. Participating practices had a project module installed in their electronic medical record system, and GPs were encouraged to perform diagnostic coding during consultations with LBP patients. The ICPC-2 diagnostic codes L02, L03, L84, and L86 triggered a study-specific pop-up in the electronic patient record. If a patient met the inclusion criteria, the GP invited the patient to participate; however, the GP could also choose not to include the patient in the study. If the patient accepted participation, consent was given by filling in the project pop-up. Patients consenting to participate were informed that completing the questionnaire was not a requirement for study participation, but they were encouraged to do so. Patients could discontinue study participation at any time and without any consequences for their treatment. The inclusion criteria were consulting a GP for LBP of any duration for the first time within 3 months, with or without associated radiculopathy, and aged 18 to 65 years. The exclusion criteria were pregnancy, insufficient language skills to complete the questionnaires in Danish, and serious underlying disease (e.g. signs of fracture, cauda equina syndrome, malignancy, osteoporosis, or spinal inflammatory arthritis). The serious underlying diseases were not within the scope of this study, as referral of patients with these conditions is generally considered appropriate.

### Randomisation and blinding

Practices were randomised 1:1 to the MuIS or PaIS. Practices were stratified by list size (≤2000 patients; 2001 to 5000 patients, or >5000 patients) in random permuted blocks of two, four, and six. Randomisation was performed by the statistical group at Aalborg University Hospital using Stata (SE version 11.2, College Station, TX, USA) with the Stata module RALLOC to generate the allocation sequence. After a practice signed up for participation, the coordinating secretary or the secretary’s assistant assigned it a participation number and opened the corresponding sealed envelope with the allocation information. The envelopes were kept at the secretary’s office, and information regarding the allocation sequence was kept in a locked safe-box until the analysis was finished. The analysis group (AR, CEJ, and MDB) was blinded to the allocation status. Patients knew that they were participating in a study, but they were not informed of their allocation status. Allocation was not blinded for the general practice personnel, the outreach visitors, the researcher (MBJ) guiding the outreach visitors, and other technical staff in contact with the practices and outreach visitors, but they were all informed that the analysis group was blinded.

### Procedures

The MuIS practices and the PaIS practices were offered usual implementation activities, which included an invitation to a regional information meeting about the guidelines, information about the guidelines described in a newsletter from The Quality Unit for General Practice in the North Denmark Region, and the opportunity to learn about the new guidelines with peers as part of the GPs’ continuing medical education. Furthermore, all practices were offered project-related passive activities, which included a referral opportunity for patients with psycho-social problems, guideline-concordant structured computerised medical record pop-ups, financial incentives to participate, and reminders about project activities.

In addition, the MuIS practices had outreach visits (before including patients) by primary care physiotherapists who were specially trained to convey the content of the LBP guidelines to GPs [9]. During the study, the MuIS practices were offered follow-up contacts with the outreach visitor. After each practice contact, the outreach visitor registered which topics had been discussed and the duration of the contact. The MuIS practices were also offered quality reports to reflect upon their treatment of LBP (e.g. how many patients they referred and how often they made new appointments for patients with LBP) and risk stratification tools integrated into the electronic medical record system (STarT Back Tool and screening questions regarding psycho-social risk factors) [9]. The STarT Back Tool subgroups patients according to prognosis and guides treatment pathways [11]. However, the physiotherapists that the GPs could refer to were not offered any special training programme for this study, i.e. they did not receive the training programme as delivered in the British randomised STarT Back Trial [11]. GPs in the MuIS group had the STarT Back Tool embedded into a pop-up in their electronic medical record system and could use the STarT Back Tool to guide treatment as well as give this information to the physiotherapists when referring a patient. Additional questions in the pop-up addressed psycho-social barriers to recovery, such as pending claims or work-related problems [9]. These risk stratification tools could be used at the initial consultation or be used if the patient had not improved after 2 weeks with LBP.

All patients (regardless of allocation group) were invited to fill in a questionnaire at home after the initial consultation. They could either complete questionnaires on the Internet or fill in and return paper versions. The questionnaires were typically filled in on the date of inclusion. The questionnaire included the STarT Back Tool and other patient-reported outcomes (see the “Clinical outcomes” section). This information was collected by the researchers and no information or feedback regarding the patients’ self-reported STarT Back questionnaire and the other patient-reported outcomes were given to the GP.

Follow-up questionnaires were sent to the patient after 4, 8, and 52 weeks; in case of no response, a reminder was sent after 14 days. If a patient was seen again in general practice due to a LBP diagnosis within 3 months after inclusion, the pop-up appeared again and the GP was thereby prompted to fill in further information about the patient [9].

GPs in the PaIS group received incentives of 1500 DKK (~£150), and GPs in the MuIS group received 2500 DKK (~£250) for study participation. This covered downloading and installing software to restructure their electronic medical records, for time spent including patients, for receiving outreach visits, and for the on-going contact with the outreach visitors. Participating practices were contacted once every fortnight to remind them of patient inclusion. Reminders were given in the form of emails, as postal letters, in newsletters for GPs on the internet, or in local newspaper articles. On a few occasions, the reminder included a friendly remark on remembering to include patients but in most cases, the practices were just informed about the study and thanked for their participation. The North Denmark Region’s administration provided data for the primary outcome, which included referrals to Danish hospitals for LBP and data on healthcare utilisation and unit costs (Additional file 1). Questionnaire data were kept and merged by an external data manager at the North Denmark Region’s Department of Information Technology. Data were merged using the unique personal identification number assigned to all Danish citizens.

### Clinical outcomes

Primary and secondary outcomes pertained to the patient. The primary outcome was the referral of patients to secondary healthcare within 12 weeks with an LBP code (ICD10 codes DM 40–43, DM 45–49, DM 51, DM 53–54, DM 95–96, and DM 99). Post hoc, it was decided to run a sensitivity analysis with follow-up after 8, 16, and 52 weeks. Secondary outcomes were assessed using patient-reported questionnaires after 4, 8, and 52 weeks. The secondary outcomes were employment status (y/n); sick leave within the last 28 days (y/n) measured after 4 and 8 weeks; sick leave within the last 14 days (y/n) measured after 52 weeks; satisfaction with the received treatment, which was categorised on a visual analogue scale (VAS) in two groups (0–5 or 6–10, higher score indicating more satisfaction); and satisfaction with the treatment outcome, which was categorised on a VAS in two groups (0–5 or 6–10, higher score indicating more satisfaction). The questions regarding satisfaction were formulated as “To what extent are you satisfied with the treatment you have received for your back problems?” and “To what extent are you satisfied with the results from your back treatment?” Patients responded by selecting from 11-point numerical rating scales. The scales were labelled at 0 (highly unsatisfied) and at 10 (highly satisfied). Other secondary outcomes were functional disability, assessed by the Roland Morris Disability Questionnaire (RMDQ) [12] (Patrick version, scale 0–23 points, higher scores indicating more disability); back pain intensity assessed by a numerical rating scale (0–10 points, higher scores indicating more intense pain); and general health assessed by the EuroQol visual analogue scale (EQ VAS; 0–100 points, higher score indicating a higher self-rated general health). The combined set of primary and secondary outcomes is in line with recommendations for a core set of outcomes in LBP studies [13].

The data we collected via pop-ups were stored in a national database for general practice. This included referrals registered by the GPs, duration of pain, GPs’ assessment of improvement in patients’ symptoms, as well as tertiary outcomes which included a description of how many patients were scored with The STarT Back Tool by the GPs, how often the GPs assessed their quality report, and a process evaluation (tertiary outcome) of GPs’ skills, beliefs, and behaviours and the patients’ beliefs and behaviours [9]. The national database closed due to legal issues and data collected via pop-ups were lost. Therefore, the primary outcome was assessed using data on referrals provided by the North Denmark Region’s administration. Data on pain duration were, however, lost together with data on tertiary outcomes [9]. This shift in the data source for the primary outcome was made during the recruitment stage and while the assessors were still blinded.

### Cost-effectiveness analysis

Alongside this cluster randomised controlled trial, an economic evaluation was conducted to assess whether it was cost effective to implement the MuIS. Costs collected from a healthcare sector perspective and the probability of not being referred to secondary care within 12 weeks were used as outcome measures in a cost-effectiveness analysis. The evaluation was carried out as a within-trial assessment with a 12-week time horizon [14]. The Danish National Health Insurance Service Register, the Danish National Patient Register, and the Danish National Prescription Registry provided individual level data on resource utilisation and unit costs (Additional file 1). The following costs were included: primary and secondary healthcare costs, publicly subsidised costs of medicine, and intervention costs [15]. Costs were adjusted for differential timing using the consumer price index provided by Statistics Denmark and presented in British pounds (£) for the year 2015.

The base case analysis considered costs and effects adjusted for patients’ age, patients’ sex, and practice size. Costs were furthermore adjusted for baseline healthcare costs within 12 months prior to inclusion. A generalised linear model with a gamma family was applied for the cost regression, while a logistic regression was applied for the effectiveness measure. For the base case analysis, a probabilistic sensitivity analysis with 5000 bootstrap replications was conducted and this illustrates the combined uncertainty around the decision to adopt the MuIS (Fig. 2). The unadjusted results are presented in a scenario analysis.

### Statistical analysis

A power calculation was performed to detect a between-group difference of 5 % in referrals to secondary healthcare within 12 weeks—13 % in the MuIS group and 18 % in the PaIS group. We expected to recruit 100 practices with an unequal cluster size [9]. The sample size was estimated with 90 % power and a 5 % level of confidence. Based on a conservative estimate of a likely cluster effect of 16 %, we needed to include 2700 patients [9]. The recruitment coincided with a conflict between the Danish regions and the Organization of General Practitioners in Denmark, and this was likely to have affected GPs’ willingness to participate. Consequently, after 15 months, it was decided to accept the inclusion of 60 practices. This decision was made before data on the primary outcome were collected and while the assessors remained blinded. Descriptive statistics included the number (%) for categorical variables and mean (SD) or median (IQRs) for continuous variables, depending on the distribution of the variable. However, costs are presented as means (SD) even though distribution of costs was right skewed (Additional file 1). Differences in baseline characteristics between the two study groups were analysed using Fisher’s exact test for categorical variables and the two-sample *t* test or the Mann-Whitney *U* test for continuous variables. For the primary outcome (referral to secondary healthcare) and the secondary binary outcomes (employment status, sick leave, satisfaction with received treatment, and satisfaction with treatment outcome), the odds ratios between the two groups were estimated using a generalised estimating equation (GEE) model with logit link and an interchangeable correlation to model the within-practice correlation. The continuous outcomes (RDMQ, back pain intensity, and EQ VAS) were analysed using linear mixed effects models with groups and weeks from baseline as fixed effects and patients within practices as nested random effects. The fixed effects were modelled as an interaction term between the groups and weeks from baseline. Results were presented unadjusted and adjusted for patients’ age (restricted cubic spline), sex (binary), and practice size (restricted cubic spline) [16]. Using the method described by Wu et al., the within-cluster correlation for the primary outcome was estimated by an intra-class correlation coefficient [17]. An approximate confidence interval (CI) for the intra-class correlation coefficient was estimated by 1000 bootstrap replications. Throughout the analyses, 95 % CIs were reported, and a *p* value of <0.05 was considered statistically significant. Analyses were performed by the intention-to-treat principle and followed the CONSORT 2010 statement for analysing cluster randomised trials [18]. Analyses were performed using Stata (IC version 13.1) (College Station, TX, USA). This study was registered at ClinicalTrials.gov (registration number: NCT01699256).

## Results

**Fig. 1:**
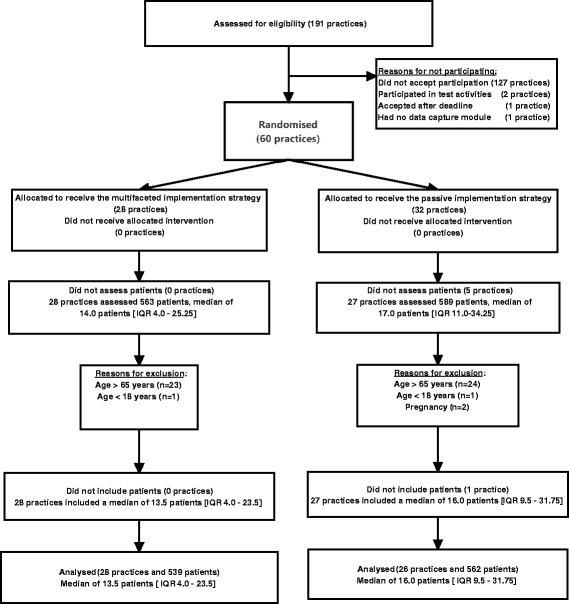
Flowchart. Sixty general practices were included in the study. A total of 55 practices (28 MuIS, 27 PaIS) assigned 1152 patients (566 MuIS, 586 PaIS) for assessment of eligibility. Fifty-four practices (28 MuIS, 26 PaIS) included and contributed with 1101 patients to the analysis for the primary outcome

**Fig. 2:**
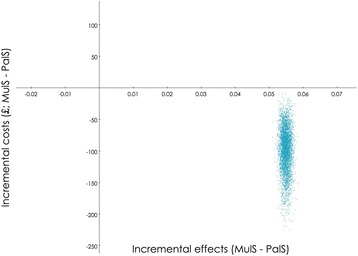
Probabilistic sensitivity analysis. A probabilistic sensitivity analysis with 5000 bootstrap replications confirms that the MuIS is cost effective compared to the PaIS. The effects are calculated as the probability of not being referred in the MuIS group minus the probability of not being referred in the PaIS group

Practices were enrolled between January 22, 2013 and March 15, 2014, and recruited patients until June 30, 2014 with a subsequent 1-year follow-up until June 29, 2015. Of the 60 general practices recruited, 28 were randomised to the MuIS group and 32 were randomised to the PaIS group (Table 1). Six practices that did not include patients were lost to follow-up. Fifty-four (90 %) practices (28 MuIS, 26 PaIS) included a total of 1101 patients (Fig. 1). Follow-up data for the primary outcome were available for 1101 (100 %) of the patients. Patients had an average age of 43 (SD 12.0) years, and 550 (49.8 %) patients were women (Table 1). Twenty-seven patients (5.0 %) in the MuIS group were referred to secondary care within 12 weeks vs. 59 patients (10.5 %) in the PaIS group (OR 0.52, 95 % CI 0.29 to 0.93; *p* = 0.027). The estimate was similar when adjusting for patients’ age, patients’ sex, and practice size (AOR 0.52, 95 % CI 0.30 to 0.90; *p* = 0.020) (Table 2). In a sensitivity analysis of the primary outcome with follow-ups after 8, 16, and 52 weeks, the estimates were not significantly changed. The intra-class correlation for the primary outcome was 0.015 (95 % CI_approximate_ 0.011 to 0.069). There were no statistically significant differences in effect on employment status, sick leave, functional status, and EQ VAS between the two groups, but patients in the PaIS group were significantly more satisfied with the treatment outcome than patients in the MuIS group (Table 2). In a base case analysis, the MuIS dominated the PaIS with higher effects achieved at a lower cost of £−52.47 (95 % CI −141.24 to 36.30). The probabilistic sensitivity analysis confirmed the dominance of the MuIS (Fig. 2). The scenario analysis also found the MuIS to be dominant with a cost saving of £−93.20 (95 % CI −198.38 to 11.99 per patient). Details of the healthcare utilisation and costs are provided in Additional file 1. As part of the intervention, all practices in the MuIS group had at least one outreach visit with a median duration of 60 min [IQR 60 to 76.25]; the median time spent on follow-up (visits or phone calls) was 60 min [IQR 37.5 to 60]. At every initial visit, practices were represented by GPs, and in five cases (17.9 %), GP trainees also participated. Discussions about the clinical examination, triage, coding of patients with LBP, general advice, importance of making follow-up appointments, the STarT Back Tool, questions regarding psycho-social risk factors, referral in primary healthcare, and the handing-out of written material were undertaken at 28 initial visits (100 %). Discussions regarding patient history and referrals to secondary healthcare were undertaken at 27 (96.4 %) of the initial visits, and instructions on the use of the computer programme were provided at 13 (46.4 %) of the initial visits. When comparing participating practices and non-participating practices in the North Denmark Region prior to this study (October 2011), we found that a similar proportion of patients in these practices were referred to secondary care with back-specific diagnoses (4.9 ‰ IQR 3.88 to 6.48 vs. 5.2 ‰ IQR 3.10 to 6.97, *p* = 0.818). Moreover, participating and non-participating practices were of a similar size (median 2061.5 patients [IQR 1636 to 3876] vs. 2227 patients [1642 to 3888], *p* = 0.957). Among participating practices, 42 (70 %) had a medical outreach visit from the Quality Unit for General Practice in the North Denmark Region in 2011 compared with 72 (55 %) among non-participating practices (*p* = 0.057).

**Table 1:** Baseline characteristics

	MuIS group	PaIS group
	(*N* = 28 practices, *n* = 539 patients)	(*N* = 32 practices, *n* = 562 patients)
Practice characteristics		
Practice size (number of listed patients)	1883 [IQR 1602 to 3475]	2086 [IQR 1649 to 3876]
Medical outreach visit in 2011 (yes)	20 (71.4 %)	22 (68.8 %)
Referral rate in 2011 (‰)^a^	4.4 [IQR 3.0 to 6.0]	4.9 [IQR 3.9 to 6.5]
Patient characteristics		
Age (years)^b^	43.8 (11.8)	42.6 (12.1)
Sex (male)^b^	282 (52.3 %)	272 (48.1 %)
Education at college level or more (yes)^c^	58 (27.2 %)	53 (21.4 %)
Co-morbidity (yes)^c^	85 (39.9 %)	86 (35.5 %)
Employed or self-employed (yes)^c^	159 (74.0 %)	187 (75.1 %)
Sick leave, LBP-related during the last 2 weeks (yes)^c^	100 (56.2 %)	118 (54.4 %)
Roland Morris Disability Questionnaire (0–23 points)^c^	14.2 (5.5)	13.0 (5.8)
Back pain intensity (0–10 points)^c^	6.2 (2.1)	6.1 (2.2)
EQ VAS (0–100 points)^c^	54.4 (22.8)	55.6 (22.4)
STarT Back Tool score (low risk)^c^	51 (25.0 %)	73 (30.8 %)
STarT Back Tool score (medium risk)^c^	89 (43.6 %)	87 (36.7 %)
STarT Back Tool score (high risk)^c^	64 (31.4 %)	77 (32.5 %)

Data are presented as the median [IQR], mean (SD), or *n* (%)

^a^Referral rate to secondary healthcare is calculated by the total number of listed patients in the practice (including patients without back pain) divided by the number of these patients referred to secondary healthcare with a back diagnosis in 2011

^b^Baseline data collected by the GP at the initial consultation. Information regarding age and sex was available in 1101 (100 %) patients, representing 54 (90.0 %) of the included practices

^c^Data collected via questionnaires after the initial consultation (*n* = 475, representing 50 practices). Complete data was available for baseline characteristics for practices. However, completeness for the patient reported measures varied education level (*n* = 461), co-morbidity (*n* = 455), employment status (*n* = 464), sick leave (*n* = 395), Roland Morris Disability score (*n* = 406), back pain intensity (*n* = 457), EQ-5D VAS (*n* = 466), and STarT Back Tool (*n* = 441)

**Table 2:** Results

	MuIS group^a^	PaIS group^a^	OR^b^	*p* value	AOR^b^	*p* value
Referral to secondary healthcare (y)						
After 12 weeks	27 (5.0 %)	59 (10.5 %)	0.52 (0.29 to 0.93)	0.027	0.52 (0.30 to 0.90)	0.020
Employment status (y)						
After 4 weeks	113 (74.8 %)	117 (73.1 %)	1.18 (0.67 to 2.08)	0.563	1.26 (0.71 to 2.24)	0.424
After 8 weeks	111 (77.1 %)	124 (72.1 %)	1.36 (0.76 to 2.43)	0.297	1.42 (0.89 to 2.26)	0.141
After 52 weeks	101 (71.1 %)	109 (71.2 %)	1.02 (0.60 to 1.74)	0.947	0.95 (0.55 to 1.63)	0.850
Sick leave within 14 days (y)						
After 4 weeks	54 (42.9 %)	60 (46.2 %)	0.87 (0.55 to 1.40)	0.577	0.90 (0.57 to 1.43)	0.658
After 8 weeks	32 (25.4 %)	43 (29.5 %)	0.82 (0.44 to 1.53)	0.533	0.84 (0.44 to 1.61)	0.605
After 52 weeks	17 (13.7 %)	19 (14.8 %)	1.00 (0.59 to 1.73)	0.981	0.97 (0.52 to 1.82)	0.922
Satisfaction with received treatment (y)						
After 4 weeks	83 (56.5 %)	99 (64.3 %)	0.72 (0.48 to 1.07)	0.105	0.75 (0.53 to 1.07)	0.112
After 8 weeks	81 (57.9 %)	114 (68.3 %)	0.64 (0.41 to 0.99)	0.046	0.66 (0.43 to 1.02)	0.061
After 52 weeks	85 (57.8 %)	105 (68.6 %)	0.62 (0.39 to 0.98)	0.040	0.61 (0.39 to 0.95)	0.029
Satisfaction with treatment outcome (y)						
After 4 weeks	71 (48.3 %)	82 (56.2 %)	0.68 (0.47 to 0.98)	0.037	0.72 (0.51 to 1.00)	0.050
After 8 weeks	69 (49.3 %)	98 (60.1 %)	0.64 (0.39 to 1.04)	0.073	0.66 (0.42 to 1.05)	0.081
After 52 weeks	75 (51.0 %)	102 (67.1 %)	0.51 (0.32 to 0.84)	0.007	0.50 (0.31 to 0.81)	0.004
RMDQ (0–23 points)			Unadjusted difference		Adjusted difference	
Dif 4 weeks—baseline	−4.23	−2.81	−1.42 (−2.88 to 0.39)	0.056	−1.34 (2.77 to 0.09)	0.067
Dif 8 weeks—baseline	−5.73	−4.59	−1.14 (−2.59 to 0.30)	0.121	−1.26 (−2.68 to 0.16)	0.083
Dif 52 weeks—baseline	−7.16	−6.50	−0.67 (−2.13 to 0.80)	0.373	−0.74 (−2.18 to 0.70)	0.316
Back pain intensity (0–10 points)						
Dif 4 weeks—baseline	−1.96	−1.54	−0.42 (−1.02 to 0.19)	0.176	−0.53 (−1.12 to 0.69)	0.083
Dif 8 weeks—baseline	−2.29	−2.31	0.03 (−0.57 to 0.63)	0.931	0.01 (−0.57 to 0.60)	0.972
Dif 52 weeks—baseline	−2.43	−2.77	0.33 (−0.27 to 0.93)	0.282	0.29 (−0.30 to 0.89)	0.328
EQ VAS (0–100 points)						
Dif 4 weeks—baseline	10.57	8.78	1.79 (−4.13 to 7.71)	0.553	2.96 (−2.51 to 8.43)	0.288
Dif 8 weeks—baseline	15.90	13.84	2.06 (−3.83 to 7.95)	0.493	2.46 (−2.95 to 7.87)	0.374
Dif 52 weeks—baseline	15.46	14.89	0.58 (−5.34 to 6.50)	0.848	1.25 (−4.20 to 6.70)	0.653

The primary outcome (referral to secondary healthcare) was measured after 12 weeks and was collected from registries with a 100 % follow-up rate, representing 54 practices. The intra-class correlation for the primary outcome was 0.015 (95 % CI_approximate_ 0.011 to 0.069). The secondary outcomes (employment status, sick leave, satisfaction with received treatment, satisfaction with treatment results, Roland Morris Disability Questionnaire (RMDQ), back pain measured by numerical pain rating [back pain intensity], and EuroQol VAS (EQ VAS)) were measured after 4, 8, and 52 weeks. All secondary outcomes were collected from patient questionnaires. A total of 475 patients, representing 50 (83.3 %) practices, participated in the questionnaires

^a^Data are number (%) or differences (follow-up—baseline)

^b^Data are unadjusted odds ratios (OR 95 % CI), adjusted odds ratios (AOR 95 % CI), unadjusted mean differences (95 % CI), or adjusted mean differences (95 % CI). Adjustments are made for patients’ age, patients’ sex, and practice size

## Discussion

The MuIS changed general practice referral behaviour compared to the PaIS.

We used a combination of outcomes recommended for studies on LBP [13]. The patient-reported outcomes were validated measures, whereas questions regarding satisfaction with the received treatment and satisfaction with treatment outcomes were formulated to fit our setting. The fact that referrals to secondary healthcare could be assessed by registry data allowed a 100 % follow-up rate of the included patients for the primary outcome and for the cost-effectiveness analysis. The change in data source for the primary outcome is a limitation. Use of registry data might have introduced uncertainty in the estimates due to difference in registration practices among different hospital departments, varying completeness of registration, as well as by changes in registration practice over time. However, we found it unlikely that this should have affected the two allocation groups unevenly in our study. Hence, we did not expect bias to be introduced by the change in data source. This study was powered to recruit 2700 patients from 100 practices. The recruitment for this project unfortunately coincided with a conflict between the Danish regions and the Organization of General Practitioners in Denmark, and this affected GPs’ willingness to participate. Consequently, after 15 months, the inclusion of 60 practices and approximately 1200 patients was accepted. This decision was made while assessors were still blinded and before registry data were collected. We consider the risk of bias caused by a smaller sample very unlikely given the large effect size (5.0 vs. 10.5 %). However, generalisability might be reduced. The response rate for the secondary outcomes was low, and with responders representing 50 (83.3 %) of the practices, this may harm the validity of the secondary outcomes. The probability of referral from primary care to secondary care within 12 weeks was used as an effectiveness measure in the cost-effectiveness analysis. It is a limitation in the economic evaluation. This might be considered double counting as costs related to primary and secondary healthcare services are included in the denominator of the incremental cost-effectiveness ratio. A more appropriate effectiveness measure could be QALYs. By participating in this study, the included practices agreed to receive the intervention, which included an outreach visit while other practices might have declined participation because of resistance to elements within the MuIS. Subsequently, practices participating in the study were generally more likely to have had an outreach visit from the regional quality unit the year prior to the study compared with the non-participating practices. This might imply that the participating practices were more likely to work with quality improvement compared with non-participants.

Reviews did not offer compelling evidence for the superiority of multifaceted interventions compared to single-component interventions [19]. Several randomised controlled trials have studied the implementation of LBP clinical guidelines in general practice using different strategies [20, 24]. The use of physiotherapists as facilitators in general practice was novel in our study. A previous randomised study used outreach visits to raise the awareness of LBP guidelines in 24 centres with 2187 patients, but the management of patients was mostly unchanged by those outreach visits [20]. In another large cluster randomised controlled trial with 118 general practices and 1378 patients, the effect of two multifaceted implementation strategies was compared to a postal dissemination of guidelines. No effect on patients’ functional levels was found when using an implementation strategy including four basic education modules and flyers for patients versus postal dissemination of the guidelines. When adding motivational counselling to patients (each patient had up to three sessions of 10–15 min), a small significant difference was found in patients’ functional level, measured by the 12-item Hannover Functional Ability Questionnaire [21]. As with our study, these two studies included outreach visits at GPs’ work environments, which, in our setting, typically took place during an extended lunch break. This may have contributed to a relaxed and informal learning environment. Other studies have used workshops to implement LBP guidelines. A Dutch guideline implementation study with 67 GPs and 531 patients included a 2-h educational and clinical practice workshop in addition to a screening tool for patients with LBP and a tool for patient education. The intervention succeeded in reducing referrals from general practice to therapists (physical, exercise, or manual therapists) [22]. In the IMPLEMENT study, the researchers found a change in clinician behaviour (knowledge, attitudes, and intentions) among 92 general practices offering two facilitated interactive workshops in a total of 6 h with the purpose of decreasing X-ray referrals and increasing advice to stay active [23]. However, the change in behavioural attitude was not reflected in a difference in the actual referral rate to X-ray or CT-scan amongst GPs receiving the intervention compared with GPs receiving the usual dissemination strategy (access to guidelines) [23]. An additional study with 462 GPs and their LBP patients with accepted compensation claims were unsuccessful in improving concordance with Canadian LBP guideline recommendations. The intervention consisted of a passive knowledge transfer method that involved postal letters with guidelines and reminders [24]. In contrast, a successful intervention included a clinical decision support system as part of a multifaceted strategy, together with quality reports and peer-to-peer consultations in a large cohort study with 1200 GPs and 23,685 patients. This multifaceted strategy was found effective in reducing MRI referrals from 5.3 to 3.7 % [25]. Our study likewise found a high effect size in the rate of referral following a broad MuIS that included clinical decision support, feedback (statistics regarding LBP patients), and outreach visits. Compared with the other trials aimed at GPs, our intervention dose (consultation time with clinicians) was slightly below average. GPs in the MuIS group could use the STarT Back Tool to categorise patients into (i) low risk patients, where advice and information can stand alone, (ii) medium risk patients with extra needs for exercise treatment or manual therapy and who may benefit from a referral to physiotherapy or chiropractic treatment, or (iii) high risk patients with an additional need for addressing psycho-social barriers for recovery. This tool has been found both effective and cost effective as a tool for subgrouping LBP patients and targeting their treatment [11, 26]. The STarT Back Tool is now widely used, and as of November, 2015, it has been translated into Danish and 20 other languages [27, 28]. The STarT Back Tool was known by many physiotherapists in Denmark and was described in the Regional LBP guideline. However, training in the stratified care management programme developed at Keele University was not generally available in Denmark. Hence, to our knowledge, none of the Regions’ GPs or physiotherapists had been trained in the stratified management programme from Keele University. The GPs’ and physiotherapists’ knowledge about the STarT Back Tool has been adapted from scientific journals, from discussions with colleagues, at conferences, from the regional LBP guidelines, and, probably most importantly, from the outreach visits at the MuIS practices. In addition to the STarT Back Tool, GPs in the MuIS group had additional questions regarding psycho-social risk factors built into their electronic medical record system regarding work problems, compensation claims, and psychological or social barriers to recovery. The tools available to the GPs may be viewed in the context of the coloured flags [3]. In combination, the STarT Back Tool and the questions about psycho-social risk factors incorporate biological, psychological, and social aspects. Patients with red flags (serious pathology) were excluded from our study. Yellow flags (beliefs, emotional responses, and pain behaviour) were addressed by the STarT Back Tool. Blue flags (perceptions about the relationship between work and health), black flags (rehabilitation/compensation system or contextual obstacles, such as legislation and injury claim conflicts), and orange flags (psychiatric factors) were encompassed by the additional questions regarding psycho-social problems. Satisfaction was planned to be analysed as a continuous variable but a non-parametric distribution of data led us to dichotomise the two satisfaction variables and this may have led to loss of information regarding satisfaction. We found a reduced satisfaction among patients in the MuIS group. This discrepancy between functional outcome measures and patients’ satisfaction has also been reported by Takeyachi et al. [29]. We do not know why patients in the MuIS group were more dissatisfied than patients in the PaIS group. One reason could be GPs advice to stay active regardless of patients’ pain, or that patients with low risk were recommended minimum treatment, or it could be related to unfulfilled expectations induced by GPs’ information of an expected good prognosis.

Our study adds the following new knowledge to existing literature: a multifaceted implementation strategy can significantly change the referral behaviour to secondary healthcare, be cost effective, without decreasing the quality of patient care. However, patients’ preferences may not necessarily support this. In the UK and many other countries, LBP is a leading healthcare burden [1, 30]. Therefore, these findings may have widespread importance for primary healthcare and policymakers.

More research into the types and doses of interventions is needed to optimise strategies for changing clinical behaviour in the future. The field of implementation science is evolving with an increasing number of possible implementation tools [5]. Passive implementation strategies, such as simply distributing a guideline might have an effect on some circumstances, and multifaceted strategies are not always superior to single-component strategies [19]. However, actively involving GPs and including a sufficient variety of components in the multifaceted strategy can change behaviour in general practice.

## Conclusions

The MuIS reduced the 12-week referral to secondary healthcare from 59 (10.5 %) to 27 (5.0 %) and was cost effective compared with the PaIS. The MuIS did not significantly change patients’ functional levels, pain levels, or self-rated health compared with the PaIS. However, patients’ satisfaction with their treatment and treatment outcomes was significantly less.

## Additional file

Additional file 1: Supplement to: Riis A, Jensen CE, Bro F, et al. A multifaceted implementation strategy versus passive implementation of low back pain guidelines in general practice: a cluster randomised controlled trial. (DOCX 19 kb)

## Abbreviations

AOR: Adjusted odds ratio
CI: Confidence interval
CRCT: Cluster randomised controlled trial
DAMD: Danish General Practice Database
EQ VAS: EuroQol visual analogue scale
GEE: Generalised estimating equation
GP: General practitioner
ICD: International Classification of Diseases
ICPC: International Classification for Primary Care
IQR: InterQuartile range
LBP: Low back pain
MuIS: Multifaceted implementation strategy
NPR: Numerical pain rating
OR: Odds ratio
PaIS: Passive implementation strategy
RMDQ: Roland Morris Disability Questionnaire
RR: Risk ratio
SD: Standard deviation
STarT: Subgrouping for targeted treatment
VAS: Visual analogue scale
