# Supplementary material for: A multifaceted implementation strategy versus passive implementation of low back pain guidelines in general practice: a cluster randomised controlled trial
Source: Implement Sci. 2016 Oct 21;11:143. doi: 10.1186/s13012-016-0509-0 (PMC5073468; doi:10.1186/s13012-016-0509-0)
Supplement: Additional file 1: — Supplement to: Riis A, Jensen CE, Bro F, et al. A multifaceted implementation strategy versus passive implementation of low back pain guidelines in general practice: a cluster randomised controlled trial. (DOCX 19 kb) [file 13012_2016_509_MOESM1_ESM.docx]

**Additional file 1**

Supplement to: Riis A, Jensen CE, Bro F, et al. A multifaceted implementation strategy versus passive implementation of low back pain guidelines in general practice: a cluster randomised controlled trial

**Costs within 12 weeks**

| Cost^a^ | Resource use (units) | | Cost (£) | |
| --- | --- | --- | --- | --- |
|  | **MuIS (n=539)** | **PaIS (n=562)** | **MuIS (n=539)** | **PaIS (n=562)** |
| Primary care |  |  |  |  |
| General practitioner | 3.81 (3.2) | 3.93 (3.7) | £85.54 (77.2) | £86.08 (94.8) |
| Physiotherapist | 4.83 (9.1) | 5.54 (9.9) | £154.71 (325.5) | £173.96 (341.8) |
| Chiropractor | 0.52 (1.8) | 0.43 (1.8) | £6.64 (31.9) | £4.28 (21.6) |
| Anaesthesiology | 0.03 (0.6) | 0.08 (0.7) | £3.18 (59.0) | £5.46 (50.9) |
| Rheumatology | 0.00 (0.0) | 0.00 (0.1) | £0.00 | £0.22 (4.8) |
| Secondary care |  |  |  |  |
| Inpatient admission | 0.01 (0.1) | 0.02 (0.1) | £38.52 (480.4) | £68.01 (653.3) |
| Outpatient clinic | 0.40 (2.1) | 0.94 (3.9) | £65.30 (322.1) | £153.93 (659.4) |
| Social medicine | 0.01 (0.1) | 0.02 (0.2) | £2.22 (44.9) | £2.99 (47.6) |
| Prescribed medications |  |  |  |  |
| Non-opioid analgesics | 0.17 (0.6) | 0.18 (0.5) | £0.37 (2.2) | £0.43 (2.6) |
| Opioid analgesics | 0.43 (1.5) | 0.45 (1.3) | £1.13 (8.4) | £1.38 (9.4) |
| Non-steroidal anti-inflammatory drugs | 0.44 (0.8) | 0.54 (0.9) | £0.42 (2.5) | £0.55 (3.5) |
| Proton pumps inhibitors | 0.12 (0.4) | 0.12 (0.4) | £0.18 (1.3) | £0.19 (1.1) |
| Anti-epileptic drugs | 0.09 (0.7) | 0.07 (0.7) | £2.30 (19.7) | £2.11 (27.0) |
| Tricyclic antidepressants | 0.04 (0.3) | 0.04 (0.2) | £0.18 (2.4) | £0.10 (1.1) |
| Intervention cost |  |  | £45.83 |  |
|  |  |  |  |  |
| Total health care cost |  |  | £406.51 (727.3) | £499.71 (1033.7) |
| Mean difference, unadjusted |  |  | £ -93.20 (-198.4 to 12.0) | |
| Mean difference, adjusted |  |  | £ -52.47 (-141.2 to 36.3) | |

*Note:* ^a^ No patient-paid costs were included. Presented are means (SD) for 1,101 patients.

Included are low back pain related primary care costs and secondary care costs from a healthcare sector perspective. Costs for developing and delivering the MuIS are included, whereas incentives for GPs to participate in research are not included. Regression analyses were applied to estimate mean incremental costs and effects. A generalised linear model with a gamma family and a square root link was applied for the cost regression. A multivariate logistic regression model was applied for the effect regression. The measure of effect was defined as the probability of not being referred to secondary care within 12 weeks. The cost and the effect regressions were adjusted for patient’s age, patient’s sex, and practice size. The cost regression was also adjusted for baseline costs (12 months before patient inclusion).
